# Glucagon‐like peptide‐1 analog improves neuronal and behavioral impairment and promotes neuroprotection in a rat model of aluminum‐induced dementia

**DOI:** 10.14814/phy2.14651

**Published:** 2020-12-23

**Authors:** Nessren M. Abd el‐Rady, Amel Ahmed, Marwa Mahmoud Abdel‐Rady, Omnia I. Ismail

**Affiliations:** ^1^ Physiology Department Faculty of Medicine Assiut University Assiut Egypt; ^2^ Department of Histology and Cell Biology Faculty of Medicine Assiut University Egypt; ^3^ Anesthesia and Intensive Care Department Faculty of Medicine Assiut University Assiut Egypt; ^4^ Department of Human Anatomy and Embryology Faculty of Medicine Assiut University Assiut Egypt

**Keywords:** aluminum‐induced dementia, Alzheimer, cytokines, eight radial arm maze, hippocampus, LIR, oxidative stress markers

## Abstract

**Background**

Alzheimer's disease (AD) is a worldwide severe medical and social burden. Liraglutide (LIR) has neuroprotective effects in preclinical animal models. Aim: To explore the probable neuroprotective impact of Glucagon‐like peptide‐1 **(**GLP‐1) on rats' behavior and to elucidate its underlying mechanisms. Methods: A total of 24 male albino rats were assigned to control, LIR (300 µg/kg subcutaneously (s.c.)), AD only (100 mg/kg aluminum chloride (AlCl_3_) orally) and LIR + AD treated groups. Eight radial arm maze was performed. Serum blood glucose, proinflammatory cytokines, oxidative stress markers were measured and hippocampal tissue homogenate neurotransmitters were evaluated. Histopathological and immunofluorescent examinations were performed. Results: LIR prevents the impairment of learning and improves both working memory and reference memory through significant reduction of serum tumor necrosis factor (TNF‐α), interleukin 6 (IL‐6) and interferon‐γ (INF‐γ) and malondialdehyde (MDA) and through the increase of superoxide dismutase (SOD), dopamine, adrenaline, and noradrenaline. LIR also improves hippocampal histological features of ALCL_3_ administrated rats and decreases the percentage of neuronal loss. Conclusion: LIR normalizes ALCL_3_‐induced dementia. It improves cognitive dysfunction and ameliorates cerebral damage.

## Graphical abstract

Neuroprotective effects of the Glucagon‐like peptide‐1 (GLP‐1) on rats’ behavior on eight radial arm maze. The underlying mechanisms via monitoring proinflammatory cytokines like tumor necrosis factor (TNF‐α), interleukin 6 (IL‐6), and interferon‐γ (INF‐γ), oxidative stress markers as malondialdehyde (MDA) & superoxide dismutase (SOD) and hippocampal tissue homogenate adrenaline, noradrenaline, and dopamine.

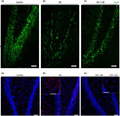

## 1 INTRODUCTION

Aluminum (Al) is a metal that is abundantly present in the earth's crust and reaches the human bodies through water and food. It is widely used in manufacturing cooking kits, antiperspirants, and drugs such as antacids (Al‐Okbi et al., 2017; Khalifa et al., 2020). Chronic administration of aluminum chloride (AlCl_3_) causes deposition of aluminum in the brain tissues that produce cognitive impairment, dementia (Chiroma et al., 2019; Shaik et al., 2019), and development of many neurodegenerative diseases including AD (Maya et al., 2016).

Alzheimer's disease is characterized by memory loss, cognitive impairment, and personality disorders accompanied by diffuse structural brain abnormalities. It is a primary worldwide medical and social affliction (Justin et al., 2016). During the disease, the senile plaques (SPs) of amyloid‐beta (A‐beta) peptides and the neurofibrillary tangles (NFTs) of the tau protein develop in specific regions of the brain, leading to the death of neuronal cells (Hardy & Selkoe, 2002). Cholinergic system in the brain, especially the basal forebrain projections to the hippocampus and cortex, is responsible for memory and learning (Cain, 1998) and is known to be affected in AD (Whitehouse et al., 1981). Noradrenaline (NA) is related to various cognitive and physiological processes, including learning and memory (Xiao et al., 2018; Zhu et al., 2018).

Glucagon‐like peptide‐1 (GLP‐1R) agonists, such as Liraglutide (LIR) and lixisenatide, are new antidiabetic products used pharmacologically to treat diabetes mellitus. They also have considerable neuroprotective effects in neurodegenerative disease, cerebral ischemia, and traumatic brain damage in animal models (Liu et al., 2015).

GLP‐1 is an endogenous incretin (insulinotropic) peptide hormone secreted from the gastrointestinal tract that plays a critical physiological role in glucose homeostasis by enhancing pancreatic insulin secretion and suppressing glucagon release and hepatic glucose output (Kim & Egan, 2008).

The hippocampal formation encompasses dentate gyrus and hippocampus proper. The dentate gyrus is a distinct structure formed of a densely compacted V‐shaped layer of tiny granule cells bundled across the hippocampus proper formed by C‐shaped Cornu Ammonis areas (Amaral et al., 2007). These areas are the CA4 (which underlies the dentate gyrus), CA3, a tiny zone called CA2, then CA1. The hippocampus is a chief component of the brain and belongs to the limbic system. It plays an imperative role in the consolidation of information from short‐term memory to long‐term memory and has a role in spatial navigation (Sheldon & Levine, 2016).

Although many studies were performed to assess the potential role of aluminum in the pathogenesis of AD (Abdelghany et al., 2019), there is still some controversy regarding the relationship between AD and Al; also, the way to prevent and/or treat AD nowadays attracts great clinical interest.

The present study was designed to evaluate the effects of Liraglutide (Victoza®) on the progression of dementia induced by the oral administration of Aluminum chloride and possible mechanisms of action by studying its potential influence on brain neurotransmitters and inflammatory and oxidative stress markers. Besides, brain tissue histopathological and immunofluorescent examinations were performed.

## 2 MATERIALS AND METHODS

### 2.1 Drugs and chemicals

Aluminum chloride was purchased from Alpha Chemika Co., India. Liraglutide (Victoza®) was purchased from Novo Nordisk S.P.A. (Rome, Italy) as pre‐filled pens containing 18 mg LIR in 3 ml of solution.

### 2.2 Experimental Animals

Twenty‐four adult male Wistar albino rats (weighing about 250 ± 20 g) were obtained from the Animal House of the Faculty of Medicine, Assiut University, Assiut, Egypt. The rats were maintained at room temperature at 22 ± 1°C with a 12‐hour dark‐light cycle and 60% humidity and kept in a well‐ventilated place for 1 week before the experimental work was begun. They were provided with food and tap water .

All the procedures used in this study were performed under the guiding principle for the Care and Use of Laboratory Animals and were approved by the ethics committee at the Faculty of Medicine, Assiut University, Assiut, Egypt.

### 2.3 Experimental design

Twenty‐four adult male albino rats were used in the experiment; randomly divided into four groups (6 rats each):

Group 1 (control group) received 0.9% NaCl solution by oral gavage.

Group 2 (LIR treated group) received 300 LIR µg/kg per day subcutaneously (s.c.) for 6 weeks (Hendarto et al., 2012).

Group 3 (AD group) AD was induced by the oral administration of a freshly prepared solution of AlCl_3_ at a dose of 100 mg/kg BW for 6 weeks (Rather et al., 2018).

Group 4 (LIR + AD treated group): LIR was given s.c as in group 2 after AD induction as in group 3.

Overnight‐fasting serum glucose levels were determined three times (at 2, 4, and 6 weeks) throughout the experiment by measuring tail vein blood glucose using a FIABiomed blood glucose‐meter (Germany). Body weights were measured three times (at 2, 4, and 6 weeks) throughout the experiment using an automated balance S/ SI‐2002 (Fisher Scientific, New York, USA).

### 2.4 Behavioral test

#### 2.4.1 Radial arm maze test

The eight‐arm Radial Arm Maze (RAM) task was performed as described before (Kim et al., 2018). Maze performance was carried out four times per day for four days and rats were allowed to make an arm choice or until 5 min have elapsed. Measurements were made using the number of working memory errors (reentering of the previously visited arm, indicated as short‐term memory) and reference memory errors (entering an unbaited arm, long‐term memory) are considered as % of the correct response. The % of the correct response was calculated by the following formula: % of correct response = number of correct response/ number of trials × 100. The latency period to enter the arm containing the food reward was recorded using a stopwatch. All the behavioral experiments were carried out from 08.00 to 01.00 p.m.

### 2.5 Collection of samples

At the end of the experiment, animals were sacrificed. Blood samples were collected, centrifuged after clotting, and the serum was separated and maintained at − 20°C until use. Brain tissues were excised and the hippocampal tissues were divided into two parts. The first part was weighed and 0.1 mol/L of perchloric acid was added (1 mg: 10 μl) and ultrasound homogenization was performed. The homogenate was centrifuged twice at 10,000 rpm/min at 4°C, and the supernatant was filtered by a microporous filter and used for the measurement of neurotransmitters by HPLC. At the same time, the remaining part was used for histological and immunohistochemical examination.

### 2.6 Biochemical parameters

1. Measurement of serum proinflammatory cytokines (TNF‐α, IL‐6, INF‐γ):
  In accordance with the pamphlet directives, the Koma Biotech ELISA kit (Cat. # K0331196 SEOUL, Korea) was used for estimating rat TNF‐α (Yousef & Hussien, 2015). IL‐6 (pg/ mg) was assessed using the rat Interleukin‐6 ELISA kit (CUSABIO, Wuhan, China), which uses the immunoassay technique of the quantitative sandwich enzyme. Solid phase sandwich ELISA (AbC 606 and AbC 607, respectively; Votrefournisseur AbCysS.A. Paris, France) measured the IFN‐γ concentrations and represented them as pg/mg.
2. Measurement of malondialdehyde (MDA) and superoxide dismutase (SOD):
  Colormetric kits supported by Biodiagnostic co (Biodiagnostic, Giza, Egypt) were approximated by MDA and SOD and expressed as nmol/ mg and U/ mg, respectively (Xia et al., 2018).
3. Measurement of hippocampal tissue homogenate adrenaline, noradrenaline, and dopamine:
  The concentrations of adrenaline, noradrenaline, and dopamine of the samples were analyzed with HPLC (Agilent Technologies 1,200 Series, G1315D DAD) according to the method of Zagrodzka et al. (Zagrodzka et al., 2000).

### 2.7 The histological techniques

#### 2.7.1 Light microscopic study

The remaining hippocampal parts were removed, fixed in Bouin's solution for 48 hr, dehydrated, embedded in paraffin and the blocks were cut serially in the coronal plane at a thickness of 5 µm. The specimens were subjected to Hematoxylin and Eosin (H&E) stain (Bolon et al., 2015).

#### 2.7.2 Immunofluorescent study

Rats were euthanized and intracardially perfused first with phosphate‐buffered saline (PBS) followed by 4% paraformaldehyde (PFA) in PBS. Brains were then post‐fixed overnight and cryoprotected with 30% sucrose at 4°C for 24–48 hr. Twenty ųm thickness sections were cut by Leica Cryostat Microsystems CM 1900‐3‐1 and left to dry for 10 minutes at Room Temperature (RT). Immunostaining was performed as previously described (Ahmed et al., 2017). Briefly, cryosections were washed three times in PBS, then blocked for 1 hour at RT with blocking solution (3% BSA and 0.2% Triton X‐100 in PBS). Anti‐NeuN antibody (ab177487; rabbit; 1:1,000; Abcam) and Recombinant Anti‐Amyloid Precursor Protein antibody (ab32136; rabbit; 1:200; Abcam) were then applied for 24 hr at 4°C followed by three rinses with PBST buffer (0.2% Triton X‐100 in PBS). Tissues were subsequently incubated with Alexa Fluor 594‐ or 488‐conjugated secondary antibodies (1:500; from Jackson ImmunoResearch, West Grove, PA) for 3 hr at RT. Hoechst 33,342 (Hst) was used to counterstain the nuclei. After triple washing with PBST buffer, the samples were mounted and cover‐slipped with anti‐fading medium (2.5% PVA‐DAPCO) and examined by Olympus “BX60F5” Fluorescence Microscope.

#### 2.7.3 Morphometric study

The cell count of the pyramidal cells in CA1 and CA3 regions were measured using computerized image analyzer system software (Leica Q 500 MCO; Leica, Wetzlar, Germany) connected to a camera attached to a Leica universal microscope at Human Anatomy and Embryology Department, Faculty of Medicine, Assiut University, Egypt.

The cell count was performed on an area of 19,259.17 μ^2^ using 400× magnification for the pyramidal cells in the mid‐portion of the CA3 field and CA1 field in all the studied groups. The percentage of neuronal loss calculated as the mean number of neurons in control sections minus the mean number of neurons in the treated group sections divided by the mean number of neurons in control sections multiplied by 100 as described by (Chiroma et al., 2019).

### 2.8 Statistical analysis

SPSS program version 16 (SPSS Inc., Chicago, USA) was used for the analysis of data. Results were expressed as mean ± *SD*. Statistical analysis was carried out using one‐way ANOVA followed by Tukey's post hoc test. Two way ANOVA test was performed between time and both body weight and blood glucose Values of *p* ≤ .05 were regarded as statistically significant (Brown, 2015).

## 3 RESULTS

### 3.1 Body weight and blood glucose measurements

**Figure 1:**
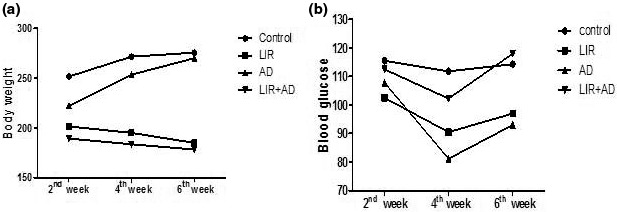
Two way ANOVA test showing the relation between time and change in body weight (a) and blood glucose (b) measured in the different studied groups. (*p* ≤ .05)

Monitoring the serum blood glucose levels in different groups showed, as expected, that the blood glucose levels in the LIR groups (LIR and AD + LIR) were significantly reduced in the second measure in comparison to the control and AD groups (Figure 1b) and this has been improved at the next measurement due to the addition of sugar to the usual diet regimen. There was a decrease in body weight (50% reduction; *p* < .001 and *p* < .01, respectively) and serum blood glucose (20% reduction; *p* < .05, *p* < .05, respectively) in both LIR group and AD + LIR group compared to the control group and AD group. Two way ANOVA indicated a highly significant effect on the blood glucose level with time (*F* (2) = 9.22, *p* < .0001). Regarding the change in body weight, LIR groups (LIR and AD + LIR) showed a significant decrease in body weight in comparison to the control groups (Two way ANOVA, *F* (3) = 16.54, *p* < .0001) (Figure 1a).

### 3.2 Eight radial arm maze

**Figure 2:**
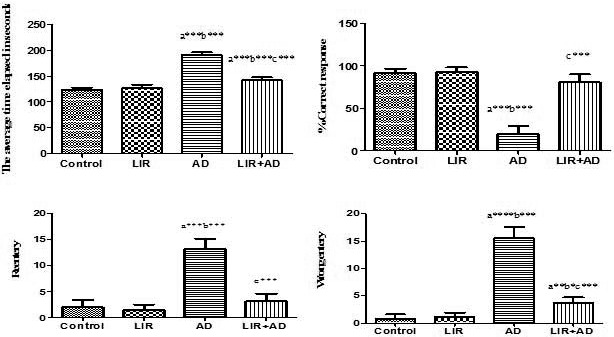
Eight radial arm maze parameters measured in the different studied groups. Data are mean ± *SD* (*n* = 6 for each group). a: control versus AD or LIR + AD, b: LIR versus AD or LIR + AD, c: AD versus LIR + AD. (*p* ≤ .05). (AD = Alzheimer's disease, LIR = liraglutide and LIR + AD = liraglutide +Alzheimer)

As shown in Figure 2, in the AD group total time was higher than that of control, with an average of 191.2 s which ultimately indicates that ALCL_3_ administration disrupts the long‐term spatial memory. There was a highly significant increase in the time elapsed (time to find the baited arm) as compared to the control animals (*p* ˂ .001). Administration of LIR causes a highly significant (*p* < .01) decrease in the elapsed time in the radial arm maze when compared to either control or AD group indicating significant memory improvement. The percentage of correct choices was significantly decreased (*p* < .001) in ALCL_3_ administrated rats when compared to the control group. Reference and working memory were assessed, respectively, by recording the number of entries into arms that were never baited (reference memory) or reentries into arms that were initially baited (working memory). The number of reference memory errors was significantly increased (*p* < .001) in the AD group. Similar to reference memory error, working memory error was also significantly increased (*p* < .001) in the AD group compared with the control group.

### 3.3 Measurement of serum proinflammatory cytokines (TNF‐α, IL‐6, and INF‐γ)

After the administration of ALCL_3_, there was moderately significant increase in serum proinflammatory cytokines (TNF‐α, IL‐6, INF‐γ) levels compared to control and LIR groups (*p* ˂ .01). But in AD + LIR rats, there was a moderately significant decrease in serum proinflammatory cytokines (TNF‐α, IL‐6, INF‐γ) levels when compared to the AD group and control group (*p* < .01) as in Table 1.

**Table 1:** Serum levels of proinflammatory cytokines (TNF‐α, IL‐6, and INF‐γ) measured in the different studied groups

Groups	TNF‐α (pg/mg)	IL‐6 (pg/mg)	INF‐γ (pg/mg)
control	99.90 ± 3.429	10.78 ± 0.1722	113.8 ± 8.448
LIR	109.4 ± 7.060	11.32 ± 0.2483	153.8 ± 3.545
AD	351.6 ± 9.975 a**b**	19.47 ± 0.4926 a**b**	453.8 ± 43.34 a**b**
LIR + AD	104.3 ± 11.48 c**	13.30 ± 2.049 a*b*c**	148.0 ± 4.147 a**b*c**

Note

Data are mean ± *SD* (*n* = 6 for each group). a: control versus AD or LIR + AD, b:LIR versus AD or LIR + AD, c: AD versus LIR + AD. (*≤0.05, **<0.01). (AD = Alzheimer's disease, LIR = liraglutide and LIR + AD = liraglutide + Alzheimer), TNF‐α = tumor necrosis factor, IL‐6 = interleukin 6, and INF‐γ = interferon‐γ.

### 3.4 Measurement of MDA and SOD

There was a moderately significant increase in the level of MDA (*p* < .01) and a moderate significant decrease in the level of SOD (*p* < .01) when both were compared to the control group. These results have been reversed in AD + LIR rats where there was a moderately significant increase in the SOD level compared to the AD group and control group (*p* < .01 and *p* < .01, respectively). But, the levels of MDA show a statically moderate decrease in AD + LIR rats when compared to the AD group and control group (Table 2).

**Table 2:** Serum levels of the measured oxidative stress markers: MDA and SOD in the different studied groups

Groups	SOD (U/mg)	MDA (nmol/mg)
Control	13.33 ± 1.870	3.930 ± 0.1049
LIR	13.12 ± 1.805	3.945 ± 0.2180
AD	7.537 ± 0.3567a**b**	5.450 ± 0.4764a**b**
LIR + AD	17.09 ± 1.245a**b**c**	4.383 ± 0.2229a**b*c**

Note

Data are mean ± *SD* (*n* = 6 for each group). a: control versus AD or LIR + AD, b: LIR versus AD or LIR + AD, c: AD versus LIR + AD. (**<0.01, ***<0.001). (AD = Alzheimer's disease, LIR = liraglutide and LIR+ AD = liraglutide +Alzheimer), MDA = malondialdehyde and SOD = superoxide dismutase (SOD).

### 3.5 Measurement of adrenaline, noradrenaline, and dopamine

**Figure 3:**
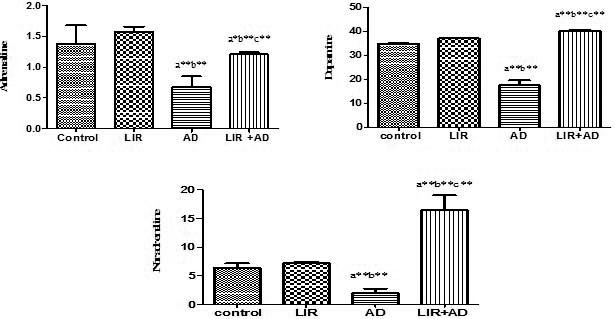
The HPLC concentrations of adrenaline, dopamine, and noradrenaline of the hippocampal tissue samples in the different studied groups. Data are mean ± *SD* (*n* = 6 for each group). a: control versus AD or LIR+ AD, b: LIR versus AD or LIR + AD, c: AD versus LIR + AD. (*p* ≤ .05). (AD = Alzheimer's disease, LIR = liraglutide and LIR + AD = liraglutide +Alzheimer)

In the present study (Figure 3), there was a moderate significant decrease in all measured neurotransmitters (dopamine, noradrenaline, and adrenaline) in the AD group when compared to the control group (*p* < .01). However, in the AD + LIR group, there was a moderately significant increase in all measured serum neurotransmitters (dopamine, adrenaline, and noradrenaline) when compared to the AD group and control group (*p* < .01).

### 3.6 The histological results

#### 3.6.1 Groups 1 and 2

**Figure 4:**
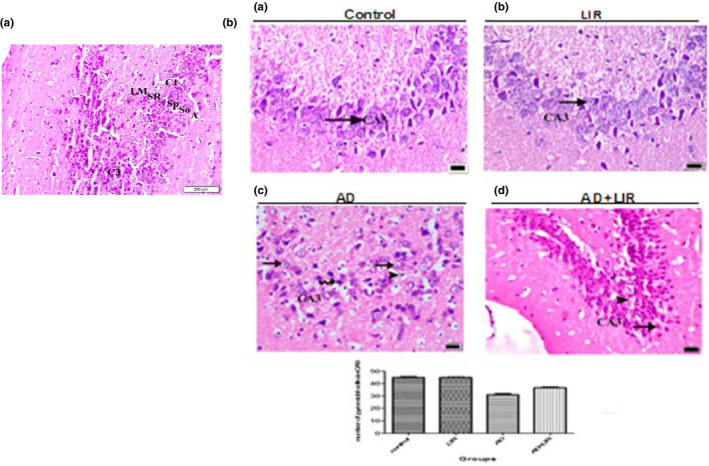
(a) A photomicrograph of the coronal section of the hippocampus of the control group shows the layers of the hippocampus which appear as follows: alveus (A), stratum oriens (SO), stratum pyramidale (SP), stratum radiatum (SR), and stratum lacunosum moleculare (LM). Note the CA1 and CA3 fields and of the hippocampus proper. (H&E x 200, Scale bar = 200 µm). (b) The photomicrographs of the coronal sections of the CA3 field of the hippocampus: (a) control group: showing that the pyramidal cells have vesicular nuclei with basophilic cytoplasm (arrow). (b) LIR group: showing the same findings as in the control group. (c) AD group: showing that many cells have dense nuclei and vacuolated cytoplasm (arrow). Other cells have dense nuclei and cytoplasm (curved arrow). Some pyknotic cells with vacuolated cytoplasm can be observed (arrow head). (d) AD + LIR received group: showing that many cells (arrow) have a normal appearance and few cells (arrow head) with vacuolated cytoplasm can be seen. (H&E x 400, Scale bar = 20 µm). The histogram shows the relation between the mean numbers of the pyramidal cells in CA3 per area of 19,259.17 μ ^2^ in different groups (AD = Alzheimer's disease, LIR = liraglutide and LIR + AD = liraglutide +Alzheimer)

The examination of the coronal sections stained by H&E of the control group and LIR treated group (results were not included) showed that the hippocampus proper was differentiated into two regions, a proximal curved region, regio inferior, toward the dentate gyrus and a distal straight one, regio superior, toward the subiculum. The regio inferior (CA3 and CA2) was composed of large‐sized pyramidal cells. In contrast, regio‐superior (CA1) was composed of small‐sized pyramidal cells (Figure 4a).

**Figure 5:**
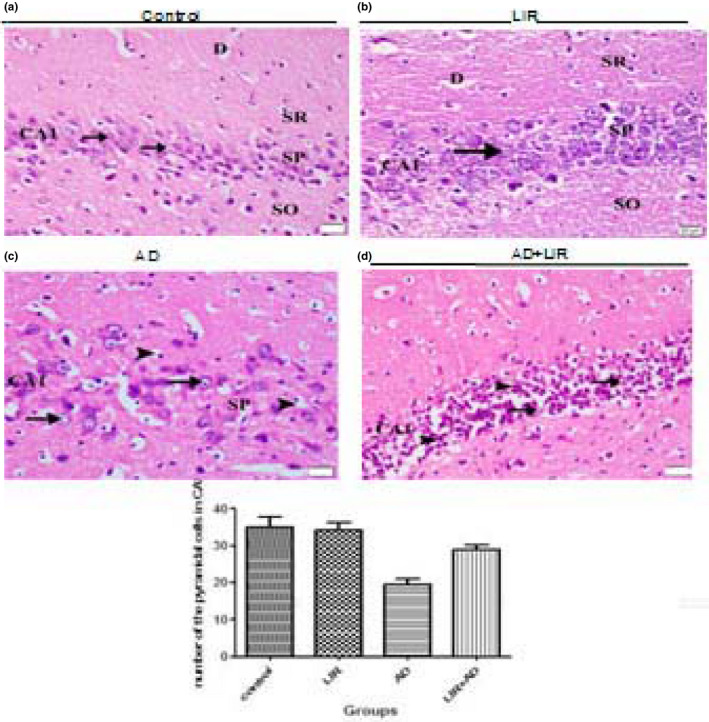
The photomicrographs of the coronal sections of the CA1 field of the hippocampus: (a) control group: showing that the pyramidal cells of the stratum pyramidale (SP) are rounded in shape with vesicular nuclei (arrow head). A prominent apical dendrite (D) emerged from the cells and passing toward stratum radiatum (SR). Note the presence of stratum radiatum superficial to the stratum pyramidale and the stratum oriens (SO) deep to it. (b) LIR group: showing the same findings as in the control group. (c) AD group: showing that most cells have dark nuclei and vacuolated cytoplasm (arrow). Some are pyknotic and surrounded by empty space (arrow head). (d) AD + LIR received group: showing that few cells (arrow) have a normal appearance and many cells (arrow head) have dense nuclei. (H&E x 400, Scale bar = 20 µm). The histogram shows the relation between the mean numbers of the pyramidal cells in CA1 per area of 19,259.17 μ ^2^ in different groups. (AD = Alzheimer's disease, LIR = liraglutide and LIR + AD = liraglutide +Alzheimer)

The pyramidal cells of the CA3 field had vesicular nuclei with basophilic cytoplasm (Figure 4b). The pyramidal cells of the CA1 field were characterized by their rounded shape with vesicular nuclei. The apices of the pyramidal cells were directed toward the stratum radiatum where their prominent apical dendrites enter, and basal dendrites passed to stratum oriens (Figure 5).

#### 3.6.2 Group 3 (AD group)

Examining the pyramidal cells in the CA3 field of the hippocampus in the AD group showed many cells with dense nuclei and vacuolated cytoplasm. Some cells had pyknotic nuclei with vacuolated cytoplasm (Figure 4b).

Light microscopic examination of the CA1 pyramidal cells revealed that the majority of these cells had darkly stained nuclei and vacuolated cytoplasm. Some cells were pyknotic and surrounded by empty space (Figure 5).

#### 3.6.3 Group 4 (AD + LIR received group)

Examining the pyramidal cells of the CA3 field of the hippocampus in the AD + LIR group showed that many cells had a normal appearance and few cells with vacuolated cytoplasm were observed (Figure 4b).

Light microscopic examination of the CA1 pyramidal cells revealed that few cells had a normal appearance, but many cells with darkly stained nuclei were noticed (Figure 5).

### 3.7 Immunofluorescent studies

**Figure 6:**
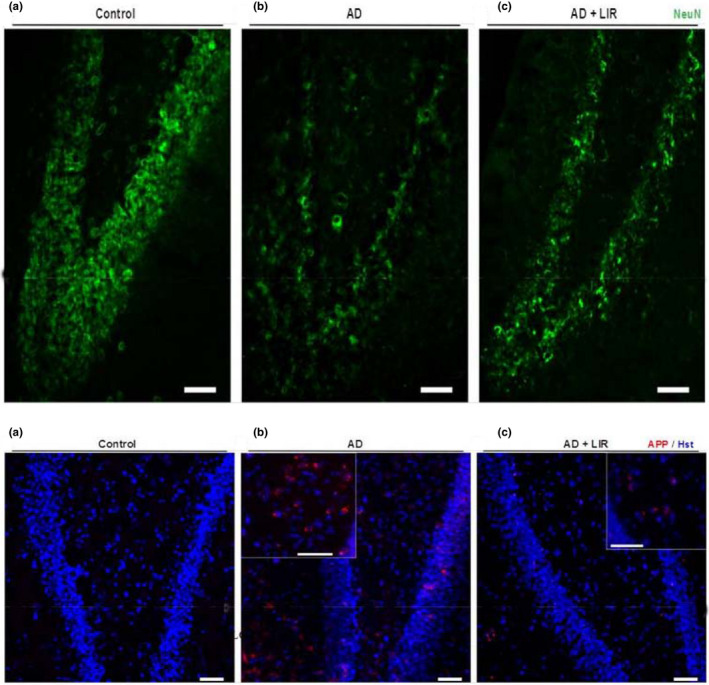
(1): Representative Immunofluorescent images of rat hippocampus immunostained for Anti‐Amyloid Precursor Protein (APP). (a) Coronal section of the albino rat shows negative staining for APP. (b) Apparent deposition of APP in the AD group. (c) AD + LIR group shows a sparse deposition of APP. (*Insets*) Higher magnification of APP deposits (Scale bar = 50 µm). Figure (6; 2): Representative Immunofluorescent images of rat hippocampus immunostained for Neuronal nuclei (Anti‐NeuN). (a) Coronal section of albino rat shows numerous NeuN‐positive cells in the hippocampal proper. (b) AD group exhibits negative NeuN staining. (c) AD + LIR group restores some positive NeuN cells (Scale bar = 50 µm). (AD = Alzheimer's disease, LIR = liraglutide and LIR + AD = liraglutide +Alzheimer)

The hippocampal distribution of amyloid precursor protein (APP) was analyzed by immunohistochemistry. When compared to the control group and LIR treated group (results were not included), the AD group showed markedly enhanced APP immunostaining in the rat hippocampus. In contrast, the AD + LIR group showed the reduction of the APP‐immunoreactivity (Figure 6a).

Using NeuN immunostaining, the AD group showed decreased numbers of NeuN‐positive cells compared to the control group; however, the neurogenesis in the hippocampus proper was increased in AD + LIR rats (Figure 6b).

### 3.8 Morphometrical results

The mean number of the pyramidal cells in CA1 in the control group was found to be 35 ± 2.85 and in the LIR group was 34.25 ± 2.04. In contrast, in the AD group was 19.5 ± 1.46 and in the AD + LIR group was 29 ± 1.2 as shown in (Figure 4b). There were significant differences in the mean number of the pyramidal cells in CA1 in the hippocampus of the various rats' groups (*F* = 258.0, *p* < .0001). Tukey's post hoc revealed a decrease in the mean number of the pyramidal cells in the CA1 in the AD group compared to the control group with a mean difference of 15.5 which was statistically significant (*p* < .05). The mean number of pyramidal cells in CA1 in the AD + LIR group was increased when compared to the AD group with a mean difference of 9.5 which was statistically significant (*p* < .05). But there were no differences between control and LIR groups.

The percentage of neuronal loss in CA1in the AD group was 44.28%; however, the percentage of neuronal loss in the AD + LIR group was 17. 14%.

The mean number of the pyramidal cells in CA3 in the control group was found to be 44.90 ± 2.55 and 44.90 ± 2.04 in the LIR group. Moreover, in the AD group was 30.95 ± 3.64 and 36.70 ± 2.17 in the AD + LIR received group (Figure 5). There were significant differences in the mean number of the pyramidal cells in CA3 in the hippocampus of the various rats' groups (*F* = 129.5, *p* < .0001). Tukey's post hoc revealed a decrease in the mean number of the pyramidal cells in CA3 in the AD group when compared to the control group with a mean difference of 13.95 which was statistically significant (*p* < .05). The mean number of pyramidal cells in CA3 in the AD + LIR group was increased when compared to the AD group, with a mean difference of 5.75 which was statistically significant (*p* < .05). But there were no differences between control and LIR groups.

The percentage of neuronal loss in CA3 in the AD group was 31.06%; however, the percentage of neuronal loss in the AD + LIR group was 18. 26%.

## 4 DISCUSSION

The present study was conducted to explain the relationship between Aluminum and AD and to highlight the role of LIR in anticipating progress and exacerbating AD. Four groups were used; control, LIR, AD, and AD + LIR groups to clarify the effectiveness of LIR in reversing AlCl_3_‐induced neurotoxic cascades that appeared in behavioral, biochemical, and immunofluorescent changes in the AD model.

Alzheimer's disease (AD) is as yet an inevitable neurodegenerative issue, influencing more than 40 million individuals around the world (Zhou & Ashford, 2019). As stated by the “World Alzheimer Report 2018” (Rusek et al., 2019), there is a new case discovered every 3 seconds, mostly in low‐ and middle‐income nations. In the present study, AD was induced in Wistar rats by AlCl_3_ at a dose of 100 mg/kg (Rather et al., 2018). This is the maximum level of aluminum allowable in occupational aluminum toxicity and dialysis encephalopathy (Justin et al., 2016).

Liraglutide (LIR) (NN2211; Victoza) is a once‐daily human glucagon‐like peptide‐1 (GLP‐1) mimetic that differs from the native GLP‐1 sequence by a Lys34Arg amino‐acid substitution and addition of a C‐16 acyl chain attached at Lys26 via a glutamyl spacer (Batista et al., 2019). It crosses the blood‐brain barrier and its receptors are expressed within the brain in areas such as the hypothalamus, neocortex, and hippocampus. Even though it was approved for the treatment of diabetes, it was interestingly found to improve cognition, to enhance long‐term potentiation (LTP), and to facilitates hippocampal synaptic plasticity and cell endurance (Cork et al., 2015).

Liraglutide decreases energy intake and body weight. It is a glucoregulatory hormone that reduces the non‐fasting plasma glucose levels and increases the non‐fasting plasma insulin concentrations (Porter et al., 2010).This is in line with the present results where there was a decrease in body weight (50% reduction; *p* < .001 and *p* < .01, respectively) and serum blood glucose (20% reduction; *p* < .05, *p* < .05, respectively) in both LIR group and AD + LIR group compared to the control group and AD group.

The present study found that after aluminum chloride administration, there was a significant impairment (*p* < .001) in working memory, which was indicated by numbers of reentries of the eight radial arm maze in the AD group when compared with the control group and this was in agreement with studies (Xing et al., 2018; Zghari et al., 2018). Moreover, (Wong et al., 2016) reported that amyloid β deposition accelerates the pathogenesis of AD, decreases spatial learning, place memory, and episodic memory as well (Karthick et al., 2019).

However, when LIR is administrated after ALCL_3_, it attenuates its neurotoxic effect, prevents the impairment of learning, and improves both working memory and reference memory (the LIR + AD rats showed a highly significant decrease (*p* ˂ .001) in the average time elapsed in seconds when compared to AD group) giving a great hope that LIR can be used in human too as a potential treatment for AD.

In the present study, the degenerative changes in the neurons of the hippocampus obtained from AD rats were widely noticed by light microscopic examination. These findings might explain the memory deficits observed in this work after aluminum administration. These results were in agreement with (Abdelghany et al., 2019) study. Also, our work was in the same line with (Shaik et al., 2019), who found that large numbers of the hippocampal CA3 neurons had flame‐shaped soma and an unhealthy cellular architecture in rats that received AlCl_3_.

In the present study, the hippocampal distribution of amyloid precursor protein (APP) was analyzed by immunofluorescent study. When compared to the control group, the ALCL_3_ group showed markedly enhanced APP immunostaining in the rat hippocampus. These results could be explained by Khalifa et al. (2020), who found that AlCl_3_ exposure speeded up both amyloid‐beta‐protein generation and oligomerization.

Terry, (Terry, 2000) reported that amyloid β protein (Aβ), a consequence of APP aberrant processing, induces a neuronal loss in the brains of AD patients. Using NeuN immunostaining, ALCL_3_ administrated group showed decreased numbers of NeuN‐positive cells compared to the control group, however, the neurogenesis in the hippocampal dentate gyrus was increased in AD + LIR treated rats. These results successfully confirmed the previous pyramidal cell quantification data and indicated the neuroprotective effect of LIR in the ALCL_3_‐induced AD model. Also, the present results were in agreement with another study that concluded that aluminum citrate treatment resulted in conspicuous neuronal loss and a decrease in the numbers of NeuN + cells per field in both CA1 and CA3 of hippocampus proper (Junior et al., 2013).

The present histopathological changes could be explained by Hansen et al. (2015) study, which concluded that LIR could reach the brain, which provides a fundamental basis for direct central action of this drug. Moreover, LIR increases the neurogenesis in the dentate gyrus, neuronal activity, and density of dendritic spines and mushroom spines in hippocampal neurons (Amato & Mulè, 2019). Histopathological results strongly supported the findings obtained by biochemical and behavioral tests.

In the present work, a significant decrease in the mean numbers of the pyramidal cells in CA1 and CA3 in the AD group was found compared to the control group. The mean numbers of pyramidal cells in CA1 and CA3 in the AD + LIR group were significantly increased when compared to the AD group. But there were no differences between control and LIR groups. The percentage of neuronal loss in the AD group in CA1 and CA3 was 44.28% and 31.06%, respectively; however, the percentage of neuronal loss in the AD + LIR group was decreased and became 17.14% and 18.26%, respectively. LIR co‐administration had the capacity to decrease the neuronal loss in AD rats. The present morphometrical results were in the same line with the study of Hansen et al. (2015), who reported that Liraglutide treated mice exhibited significantly higher CA1 pyramidal neuron numbers than controls. Also, our results supported the results of Eltahawy et al. (2016) study which found that the number of neurons in the stratum pyramidale of the hippocampus was significantly less in the aluminum group than in the control group.

In this study, there was a significant increase in all measured serum proinflammatory cytokines (TNF‐α, IL‐6, INF‐γ) after ALCL_3_ administration when compared to the control group. This goes with previous studies that found a positive correlation between neuroinflammatory cytokine release and the progression of the AD, which suggests these cytokines are involved in AD pathophysiology (Alasmari et al., 2018). It has been observed that increased levels of proinflammatory cytokines may hold up phagocytosis of amyloid Aβ (Stamouli & Politis, 2016) through upregulating β‐secretase beta‐site amyloid precursor protein cleaving enzyme 1 (Alasmari et al., 2018). Aβ deposition can activate microglia and induce the production of IL‐6 and INF‐γ in the AD brain (Gubandru et al., 2013). Interestingly, the relation between AD and oxidative stress was observed in the present work and was highly evident. There was a moderately significant increase in the level of MDA (which indicates lipid peroxidation) and a moderate significant decrease in the level of SOD (which is an essential natural antioxidant) when both were compared to the control group. This goes with the previous studies which found that ALCL_3_ exposure is associated with impairment of mitochondrial functions and antioxidant defense system, in vivo and in vitro (Balgoon et al., 2019). AD stimulates macroautophagy of A*β* that may further induce cell death by destabilizing lysosomal membranes (Al‐Okbi et al., 2017).

Aluminum reduces antioxidant enzymes and increases MDA levels as a result of lipid peroxidation (Shaik et al., 2019). All these findings give a shred of growing evidence that AD is related to apoptosis either through the accumulation of Ab, which can induce neuronal apoptosis or by oxidative stress, which causes a cytochrome c release and activates caspase‐9 and caspase‐3 and overproduction of free radicals (Xing et al., 2018).

In the present study, LIR had anti‐inflammatory and anti‐oxidative activities proved by a significant decrease in serum TNF‐α, IL‐6, INF‐γ, and MDA level and the statistically significant increase in SOD. These findings were in the same line with the previous study that was found that mice treated for 4 weeks with LIR showed reductions in tumor necrosis factor‐α and interleukin‐ 6, as well as the reactive oxygen species genesis, suggesting a preventive role of LIR in inducing apoptosis (Nuzzo et al., 2019). GLP‐1 has anti‐inflammatory actions in both in vitro and in vivo models (Hölscher, 2018). It reduces microglial activation and modulates brain inflammation (Batista et al., 2019). Many studies suggested that GLP‐1 receptor agonist inhibited proinflammatory cytokine expression in cultured astrocytes (Amato & Mulè, 2019) and in natural killer cells (Iwai et al., 2014). The LIR antioxidant action might be due to the ability of LIR to act as a growth factor in the brain, increasing cell growth, proliferation, and inhibiting apoptosis (Nuzzo et al., 2019).

In the present study, there was a significant decrease in all measured neurotransmitters (dopamine, noradrenaline, and adrenaline) in the AD group (*p* value < .01). Abdelghany et al (Abdelghany et al., 2019) stated that aluminum depresses cerebrospinal fluid tetrahydrobiopterin levels which are required for the synthesis of those neurotransmitters. Nobili et al (Nobili et al., 2017) reported a decrease in hippocampal dopamine in AD patient suffering characteristic depressive episodes. Moreover, Cortés et al. (2018) noticed a gradual loss of dopaminergic neurons in an AD mouse model implicated with memory and reward dysfunction. Also, Gannon et al. (2015) stated that the loss of noradrenergic neurons and the subsequent reduction of brain noradrenaline (NA) levels are two alterations usually found in AD and usually aggravate the progression of neuroinflammation and neuronal damage (Gutiérrez et al., 2019). Regarding adrenaline, the present results go with the previous studies, which stated that AD patients have an exhibited loss of LC neuronal cells, reducing to as low as 70% in the rostral nuclei leading to reduced limbic and cortical epinephrine and norepinephrine levels (Gannon et al., 2015). Some clinics in the USA are already using adrenaline as a therapeutic option in the case of AD (Qadir et al., 2014). The use of adrenergic agonists in combination with cholinergic drugs may increase cognitive function and delays the adverse cognitive degeneracy effects associated with progressive geriatric AD (Giorgi et al., 2019).

The significant increase in all measured neurotransmitters (dopamine, adrenaline, and noradrenaline) in AD rats after the administration of LIR was noticed for the first time in this research. This result increases the beneficial use of LIR, giving the drug another tremendous clinical importance in modulating the depression and mood disturbance associated with AD.

The mechanism by which Liraglutide reduced the degenerative effects of aluminum chloride, which detected in the present work might be explained by previous studies that revealed that Glucogan‐like peptide 1 (GLP‐1) such as Liraglutide reduced the endogenous levels of ‐amyloid A in the rodent brain and amyloid plaques, preventing loss of synapses, apoptosis and enhanced associative and spatial learning. It also increased neurogenesis, decreased oxidative stress, and chronic inflammatory responses (Prakash & Kumar, 2013). It also increases the hippocampal expression of mTOR and rescues spatial memory and synaptic plasticity from Ab protein‐induced impairments in rats (Cai et al., 2014).

## 5 CONCLUSION

Chronic administration of AlCl_3_ in rats induced marked impairment of cognitive functions and histological alterations of the hippocampus. Liraglutide was able to improve cognitive dysfunction, prevent learning impairment, improve both working and reference memory and minimize cerebral damage by increasing the serum levels of inflammatory markers (TNF‐α, IL‐6, and INF‐γ), reversing the state of oxidative stress, increasing the concentrations of hippocampal tissue homogenate adrenaline, noradrenaline, and dopamine as well as preventing the histological alterations induced by AlCl_3_ and decreasing the percentage of neuronal loss, suggesting its potential role as a neuroprotectant. Such observations stand for the potential role of LIR supplementation as an adjuvant in the therapeutic strategies aimed at delaying AD disease progression and its complications, mainly the associated depression, particularly during long courses of LIR therapy.

## CONFLICT OF INTEREST

The authors have nothing to declare.

## AUTHOR CONTRIBUTIONS

Nessren M. Abd el‐Rady: conceived and designed the study, supervised conducting the experiment, shared in analyzing the data, shared in writing the paper, and critical reading the paper, submitting the paper. Marwa Mahmoud Abdel‐Rady: shared in designing the study, analyzed the data, shared in writing the paper, and critical reading the paper of the paper from the clinical point of view. Amel Ahmed and Omnia I. Ismail: shared in designing the study performed the histopathological and morphometrical studies, analyzed their results, and shared in writing the paper.

## Data Availability

Data are available upon request.
